# Side Effects Following Administration of the First Dose of Oxford-AstraZeneca’s Covishield Vaccine in Bangladesh: A Cross-Sectional Study

**DOI:** 10.3390/idr13040080

**Published:** 2021-10-11

**Authors:** Nishat Jahan, Fahad Imtiaz Rahman, Poushali Saha, Sadia Afruz Ether, ASM Roknuzzaman, Rapty Sarker, Khondoker Tashya Kalam, Kashfa Haq, Julkar Nyeen, Humayra Zaman Himi, Md. Nazmul Hossain, Mahtab Hossain Chowdhury, Mostafa Moin Uddin, Nur Haque Alam

**Affiliations:** 1 Department of Pharmacy, University of Asia Pacific, Green Road, Dhaka 1205, Bangladesh; 17203094@uap-bd.edu (A.R.); 17203089@uap-bd.edu (R.S.); 17203021@uap-bd.edu (K.T.K.); 17203078@uap-bd.edu (K.H.); 17203083@uap-bd.edu (J.N.); 17203098@uap-bd.edu (H.Z.H.); 17203114@uap-bd.edu (M.N.H.); 17203084@uap-bd.edu (M.H.C.); 2 Department of Clinical Pharmacy and Pharmacology, Faculty of Pharmacy, University of Dhaka, Dhaka 1000, Bangladesh; fahad@du.ac.bd (F.I.R.); poushali@du.ac.bd (P.S.); 3 Department of Pharmacy, Faculty of Allied Health Sciences, Daffodil International University, Dhaka 1207, Bangladesh; sadia.ph@diu.edu.bd; 4 Office of the Director General, Directorate General of Health Services (DGHS), Mohakhali, Dhaka 1212, Bangladesh; mostafamoin79@gmail.com; 5 Nutrition and Clinical Services Division (NCSD), International Centre for Diarrhoeal Disease Research, Bangladesh (icddr,b), Mohakhali, Dhaka 1000, Bangladesh; nhalam@icddrb.org

**Keywords:** COVID-19, Covishield, Oxford-AstraZeneca, post-vaccination side effects

## Abstract

In response to the raging COVID-19 pandemic, Bangladesh started its vaccine administration in early 2021; however, due to the rapid development and launch of the vaccines in the market, many people had concerns regarding the safety of these vaccines. The purpose of this study was to evaluate the side effects that were experienced by the Bangladeshi residents after receiving the first dose of the Oxford-AstraZeneca’s Covishield vaccine (ChAdOx1nCoV-19). The study was conducted using both online and printed questionnaires and the data were analysed using SPSS. The results included the responses of 474 vaccine recipients from March–April 2021. Pain at the site of injection, fever, myalgia, fatigue and headache were the most commonly reported symptoms, and the overall side effects were found to be significantly more prevalent in the younger population (*p* ≤ 0.05). These findings were consistent with the results indicated by the clinical trial of ChAdOx1nCoV-19. Logistic regression analysis further revealed that compared to people aged 70 years or above, the incidence of reported side effects was significantly higher in people aged 18–30 years (odds ratio (OR) = 8.56), 31–40 years, (OR = 5.05), 41–50 years (OR = 4.08), 51–60 years (OR = 3.77) and 61–70 years (OR = 3.67). In addition, a significantly higher percentage of female participants suffered from post-vaccination side effects compared to males (OR = 1.51). It was concluded that the Covishield vaccine was well-tolerated among people of different age groups. Nevertheless, further long-term follow-up study with a larger sample size is warranted to establish the long-term safety of the COVID-19 vaccine.

## 1. Introduction

The world has been going through a global crisis since early 2020 due to the COVID-19 pandemic, which is caused by the newly discovered coronavirus, namely, severe acute respiratory syndrome coronavirus 2 (SARS-CoV-2) [1]. After COVID-19 was first reported on 31 December 2019, in Wuhan, China [2], the World Health Organization (WHO) proclaimed the COVID-19 outbreak a public health emergency of international concern, representing a significant threat to countries with inadequate healthcare systems on 30 January 2020 [1]. The first case of COVID-19 was confirmed in Bangladesh on 8 March 2020 [3]. Following that, the government implemented several measures to prevent the disease’s spread, including a countrywide lockdown and the deployment of security forces to ensure that people maintained social distancing and followed the required safety precautions [3,4].

Although the treatment of COVID patients with broad-spectrum antibiotics and antivirals has led to the recovery of patients to some extent, many of them have faced severe adverse effects. Therefore, many pharmaceutical companies and research centers have been racing to develop safe and effective vaccinations to tackle the pandemic [5]. According to recent WHO reports, more than 210 candidate vaccines are under development, out of which, at least 48 of them are already in human trials [6].

After the arrival of the first shipment of Oxford-AstraZeneca’s Covishield vaccine (ChAdOx1nCoV-19) in Bangladesh on 25 January 2021, a countrywide COVID-19 vaccination campaign was initiated on 7 February 2021, with the target of inoculating 3.5 million people in the first month [7]. Oxford-AstraZeneca utilised a non-replicating adenoviral vector vaccine that expresses a spike protein that resembles the one produced by the SARS-CoV-2 virus following natural infection. This spike protein generated by vaccination induces a strong immune response, providing protection against COVID-19 [8]. Moreover, maintaining the cold chain all over the country is quite complicated and the adenovirus-vectored technique proved convenient since it could be kept in regular refrigerator settings, unlike Pfizer and Moderna vaccines, which require sub-zero temperatures until use [9,10].

In the UK, Brazil and the United States, phase 3 trials with ChAdOx1 nCoV-19 are being conducted to examine the vaccine’s effectiveness and safety and the interim analysis of these ongoing trials indicates that it has 70.4% efficacy against symptomatic COVID-19 after completion of a two-dose vaccination [11,12]. However, one of the most important and necessary phase 4 post-marketing adverse effect surveillance studies has yet to be reported [13].

Therefore, it is important to assess any major side effects or undesirable consequences that may occur as a result of the vaccination programs. If no serious side effects of Covishield can be established, then people with low confidence about the COVID-19 vaccine would be more willing to participate in this immunisation program, making it easier to reduce the pandemic by preventing the vulnerable population from infection and disease and stop further transmission of this disease [14,15]. With mass vaccination coverage anticipated, findings from immediate post-marketing data on COVID-19 vaccines can guide regulatory decisions and public health practice to maintain a positive benefit–risk balance [16].

The purpose of this study was to assess the short-term side effects that were experienced by the recipients of the first dose of Oxford-AstraZeneca’s Covishield vaccine in Bangladesh.

## 2. Methods

A cross-sectional study was conducted among Bangladeshi residents who were aged more than 18 years and who had received the first dose of the Covishield vaccine. To carry out this study, a questionnaire, both in English and Bengali, was developed and was made available on the Google platform. Printed copies were also used to collect data. The questionnaire was pre-tested for validity by conducting a pilot study. The respondents voluntarily participated in the study without any incentives. Each participant was allowed to provide a response only once. In some cases, multiple responses were allowed to gather data from elderly people with technical difficulties. All responses were reviewed carefully to eliminate any discrepancies. Data were collected for 1 month from 6 March to 8 April 2021.

The survey consisted of three categories of questions. The first category included the socio-demographic data of the respondents. The second category focused on the perception of the participants towards COVID-19 vaccination. The last segment comprised the vaccination-related data. The side effects were selected according to the side effects observed in the clinical trial of the ChAdOx1nCoV-19 vaccine [11].

Residents of Bangladesh who had received the first dose of the Covishield vaccine were included in the study. People who were not vaccinated against COVID-19 were excluded from the study. By using the Raosoft sample size calculator, the minimum required sample size for conducting the study was found to be 385, considering a 5% margin of error and 95% confidence interval. In the present study, in total, 474 participants who satisfied both the inclusion and exclusion criteria were purposively selected and their responses were subjected to statistical analysis.

After the data collection, it was cleaned and coded using MS Excel and statistically analysed using SPSS v. 25. All data obtained from the 474 participants were subjected to descriptive statistical analysis. Associations of the number of side effects with different demographic groups were analysed using the chi-squared (χ^2^) test. Additionally, a logistic regression analysis model was carried out via the “Enter” method to determine the influence of the different demographic characteristics on the presence of individual side effects.

The study protocol was approved by the Research Ethics Committee of the University of Asia Pacific, Bangladesh, and was conducted according to the Declarations of Helsinki. Digital scripts of informed consent were collected from each participant.

## 3. Result

### 3.1. Socio-Demographic Characteristics

**Figure 1:**
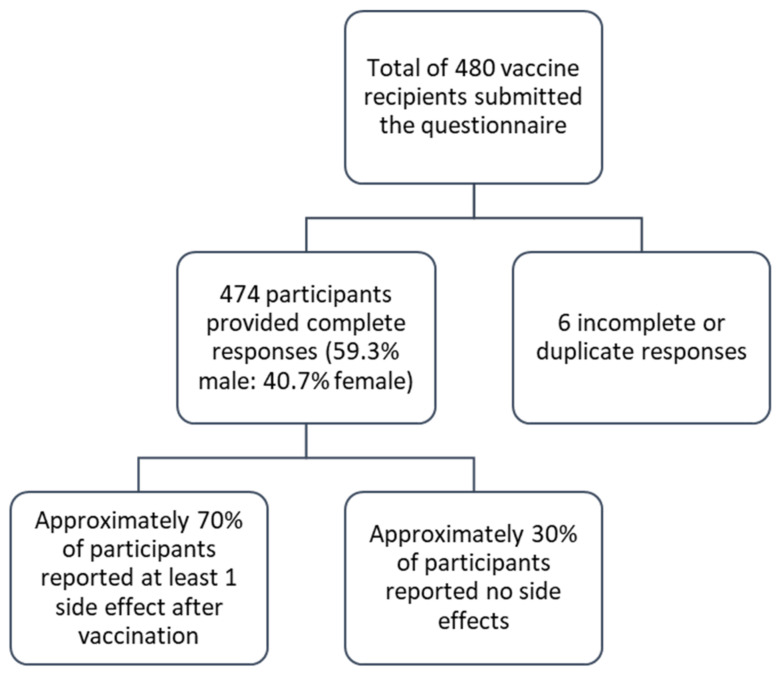
Flowchart showing the survey responses of the Covishield vaccine recipients.

All 474 participants had received the first dose of the Covishield vaccine during the initial vaccine rollout in Bangladesh. A schematic representation of the survey responses is shown in Figure 1.

The study results showed that among all the respondents, 39.9% were from rural areas and 60.1% were from urban areas of Bangladesh. Table 1 shows that the majority of participants were male (59.3%, *n* = 281) and the rest were female (40.7%, *n* = 193). Regarding the age groups, the highest number of respondents was from the 41–50-year-old group (33.3%) and the lowest number was from the 70+-year-old group (3.6%). Almost 71.3% of participants achieved university or post-university education levels. The study population comprised 34.4% that were either a teacher or a student, 20.7% were service holders, 19.6% were housewives, 12.4% were business holders, 5.1% were healthcare workers, 5.3% were unemployed and the rest included a small percentage of other occupations. Most participants received their vaccines from vaccination centres located in Dhaka (50.6%), 23.4% from Khulna and 10.8% from Rangpur (Table 1).

### 3.2. Past Medical History of Health

Most of the participants in this study reported no comorbidities (46.8%). Among the others, the majority of the respondents reported hypertension (33.3%), diabetes (24.5%), heart disease (8.4%) and lung disease (8%). About 45.1% had a history of allergic reactions to various allergens, such as the cold (19%), dust (31%), food (14.3%), insects (2.1%) and drugs (0.8%), which were associated with common symptoms, such as sneezing, coughing, itching, swelling, runny nose and shortness of breath.

**Figure 2:**
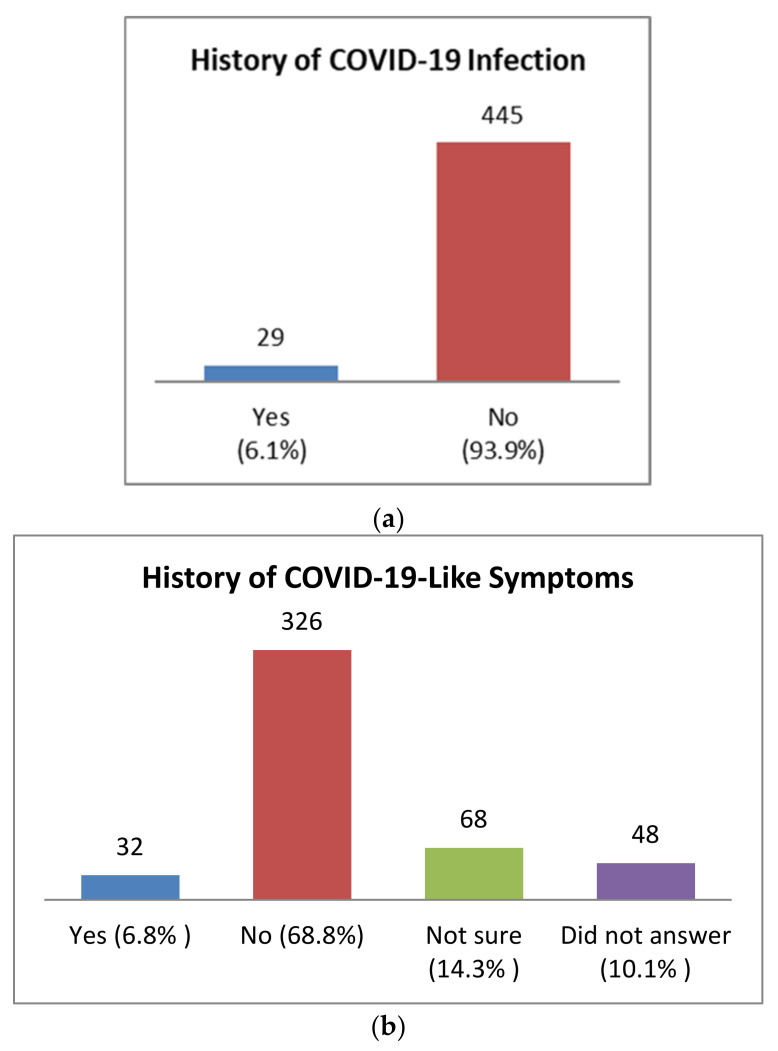
Study participant’s history of COVID-19 encounters. (**a**) Prior history of COVID-19 infection. Only cases that were confirmed with reverse transcriptase-polymerase chain reaction (RT-PCR) tests were included. (**b**) Presence of COVID-19 like symptoms before vaccination; these are cases where the study participant had one or more COVID-19-like symptoms but did not perform any confirmatory RT-PCR tests.

Among the study population, only 6.1% (*n* = 29) had a history of confirmed COVID-19 infection, while 6.8% (*n* = 32) suffered from COVID-19 like symptoms prior to vaccination (Figure 2). None of the participants reported any history of hospitalisation or plasma therapy before receiving the first dose.

It was reported that 10.8% of the participants were regular smokers and 4% were occasional smokers. Only 10% had a history of alcohol drinking, while a very small percentage of 0.2% had a previous history of recreational drug abuse.

### 3.3. Perception and Awareness of Participants Regarding AstraZeneca Covishield Vaccine

**Figure 3:**
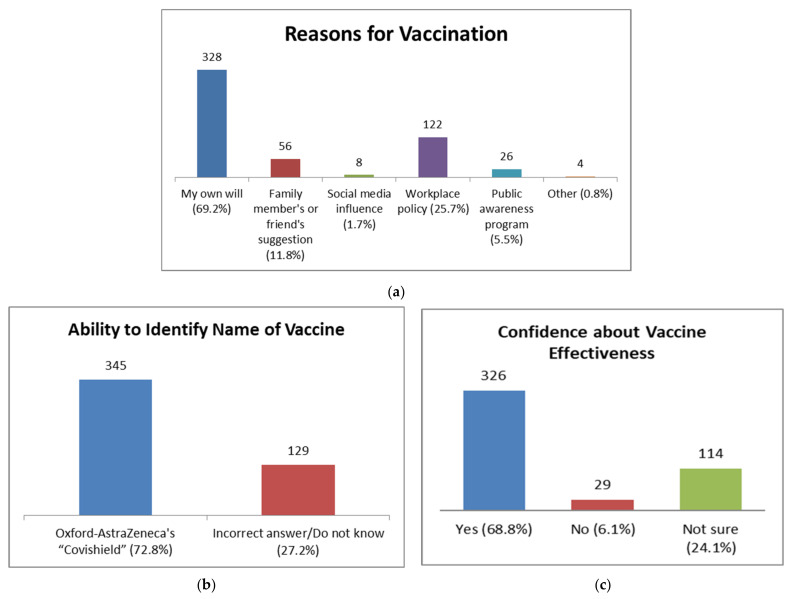
Perceptions of study participants towards COVID-19 vaccines. (**a**) Reasons for vaccination showed that the majority of participants got vaccinated due to their own will. (**b**) Most participants reported being confident about the vaccine’s effectiveness. (**c**) Ability of the vaccine recipients to identify the name of the vaccine. More than one-quarter of study participants could not correctly identify the vaccine.

Of the 474 participants, 69.2% stated that they had taken the vaccine of their own will, 25.7% due to a workplace policy, 11.8% because of a family member’s or friend’s suggestion and 5.5% were inspired by public awareness program (Figure 3A). Around 70% of vaccine recipients mentioned that they were confident regarding the efficacy of the vaccine, followed by 30% who were either not confident or not sure about it (Figure 3B). About 72.8% of vaccine recipients could identify the correct name of the vaccine that they had been administered, while 27.2% of people were not aware of it (Figure 3C).

### 3.4. Self-Reported Solicited Side Effects after Covishield Vaccination

**Figure 4:**
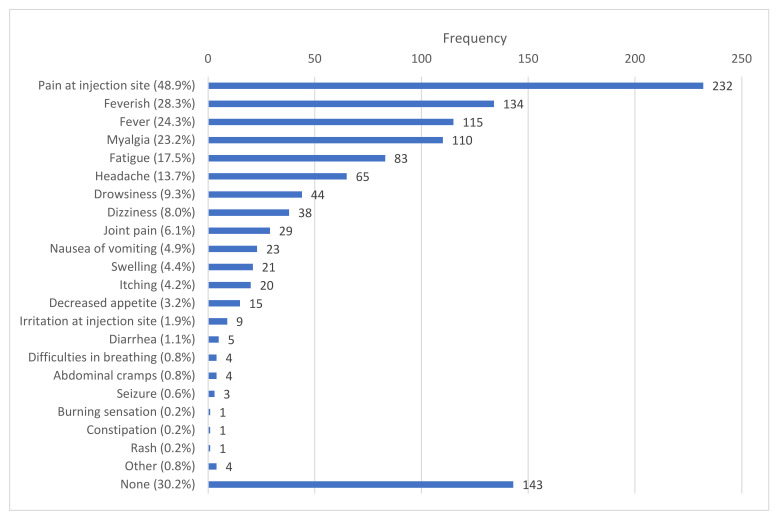
Reported side effects after the first dose of the Covishield vaccine. Pain at the injection site was the most common local side effect, while feeling feverish or having a fever seemed to be the most common systemic side effects. Thirty percent of participants reported having no side effects after their vaccination.

The solicited local side effects included mainly pain at the site of injection (48.9%, *n* = 232), swelling (4.4%, *n* = 21), itching (4.2%, *n* = 20), irritation at the site of injection (1.9%, *n* = 9) and a burning sensation (0.2%, *n* = 1). The solicited systemic side effects included fever or feeling feverish (52.6%, *n* = 249), myalgia (23.2%, *n* = 110), fatigue (17.5%, *n* = 83), a headache (13.7%, *n* = 65), drowsiness (9.3%, *n* = 44), dizziness (8.0%, *n* = 38), joint pain (6.1%, *n* = 29), nausea or vomiting (4.9%, *n* = 23), diarrhea (1.1%, *n* = 5) and a rash (0.2%, *n* = 1). A considerable percentage of vaccine recipients did not complain about any sort of side effects (30.2%, *n* = 143) (Figure 4).

**Figure 5:**
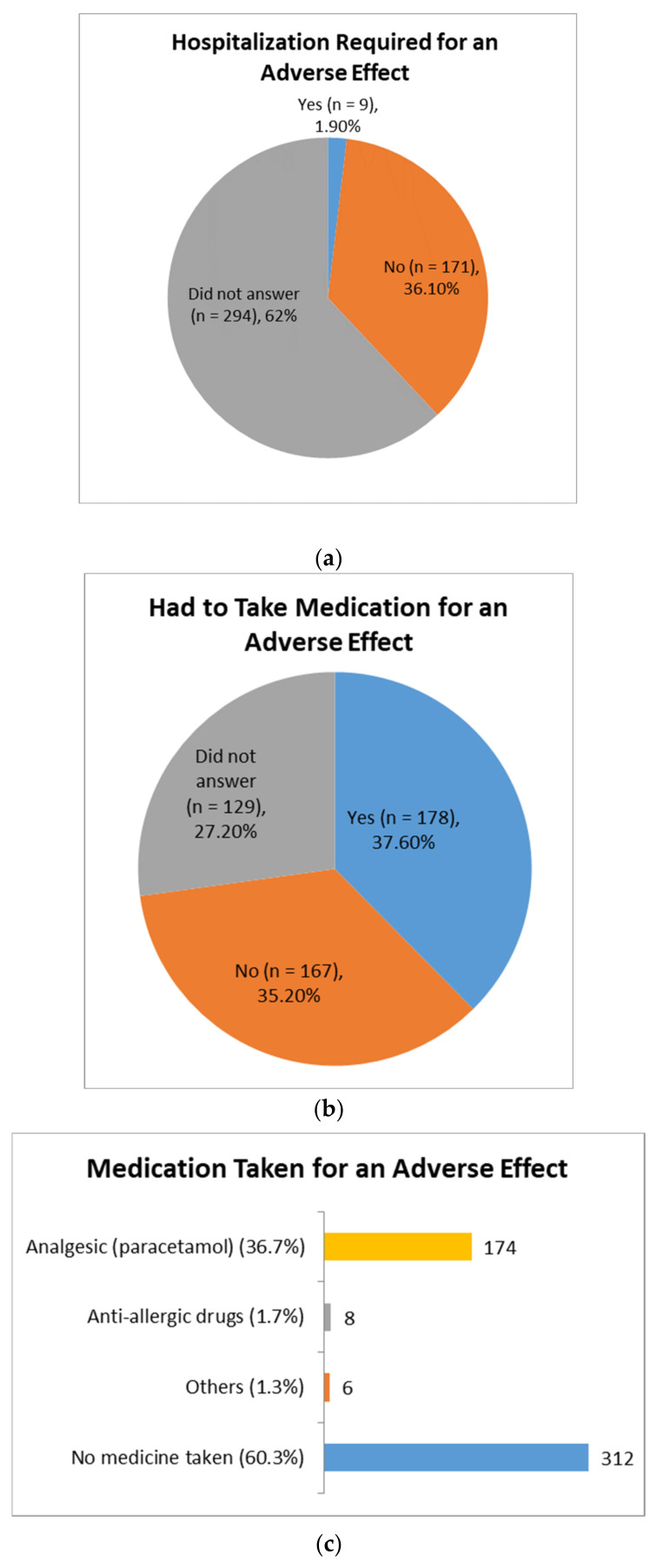
Post-vaccination adverse effect treatment of study participants. (**a**) Hospitalisation required for an adverse effect after vaccination. (**b**) Had to take medication for an adverse effect following vaccination. (**c**) Different medications taken for an adverse effect by the vaccine recipients.

After receiving the vaccine, allergic reactions were experienced by 7% of recipients, which were mainly comprised of a cold (5.5%), coughing (3.6%), fever (5.5%), itching (6.5%), swelling (2.3%) and shortness of breath (2.1%). About 37.6% of vaccine recipients had to take some sort of medication for alleviating the adverse effects. Paracetamol was the most used (36.7%), followed by antiallergic drugs (1.7%), and only 1.9% of recipients required hospitalisation (Figure 5).

### 3.5. Association between the Number of Reported COVID-19 Vaccine Side Effects and Participants’ Demographic Characteristics

A chi-squared test was performed to assess the association between the number of post-vaccination side effects and the different demographic characteristics of the participants (Table 2). A significant difference was found between the number of side effects reported by male and female participants (*p* = 0.016), where female vaccine recipients had a 1.5 times higher chance of showing side effects compared to males (*p* = 0.067) (Table 2).

The results further revealed that there was a significant difference (*p* = 0.02) between the number of side effects reported by the different age groups of participants (Table 2). Among the various age groups, approximately 37% of people aged 18–30 years reported ≥4 side effects, 61% of people aged 61–70 years reported 1–3 side effects and approximately 59% of people aged over 70 years reported no side effects in this study. The logistic regression revealed that compared to people aged more than 70 years, the odds of reporting side effects was 8.56 times higher in people aged between 18 and 30 years and 5.05 times higher in people aged between 31 and 40 years (*p* ≤ 0.05) (Table 2). This means that the ages of 18–30 years had an 8.56 times higher incidence and the ages of 31–40 years had a 5.05 times higher incidence of developing side effects than people with an age above 70 years. However, this finding would be more reliable if the study was conducted in a larger population.

No significant association was found with participants’ educational qualification, occupation type, body mass index (BMI), presence of comorbid conditions or previous history of COVID-19 infections (*p* > 0.05).

### 3.6. Association of Individual Post-Vaccination Side Effects with Gender and Age

A chi-squared test was also performed to find out the association of individual side effects with gender and age (Table 3). Fever was found to be significantly more prevalent among the 41–50-year-olds (31.6%), followed by the 31–40-year-olds (28.8%) and the 18–30-year-olds (27.1%) (*p* = 0.008). Female participants (21.8%) experienced significantly more fatigue compared to male participants (14.6%) (*p* = 0.044).

Headache, drowsiness, dizziness, nausea or vomiting and swelling were also significantly more prevalent among female participants compared to male participants (*p* ≤ 0.05). In the age group of 18–30 years, headache and drowsiness were experienced more than the other groups (*p* ≤ 0.01) (Table 3). No significant association was found among the other symptoms following vaccination.

## 4. Discussion

The unprecedented pace at which COVID-19 vaccines were developed had heightened the already existing vaccine hesitancy among general population, and further aggravated by the unregulated circulation of conspiracy theories and misinformation in the social media. Hence, research publications and information regarding the safety and efficacy of these vaccines are highly called for to prevent vaccine-related misconception and promote uptake of available vaccines [17]. After a vaccine administration, some side effects usually occur, which indicates that the vaccine is activating the body’s immune system to defend itself from the disease. These common side effects are short-lived and much less serious than developing COVID-19 or complications associated with COVID-19 [18]. The most commonly reported symptoms in this investigation were pain at the site of injection (48.9%), feeling feverish (28.3%), fever (24.3%), myalgia (23.2%), fatigue (17.5%) and headache (13.7%) (Figure 4), which are consistent with the clinical trial results of Oxford/AstraZeneca’s ChAdOx1 nCoV-19, although the frequencies of the side effects were much lower in this study. This might have happened because of differences in ethnicity, geographical location and environmental factors of the study population [19].

The study revealed that a significantly greater number of female participants suffered from post-COVID-19 vaccination side effects compared to males (*p* = 0.016). This pattern was not particularly reported in the clinical trials of Oxford/AstraZeneca. However, this is similar to the trend displayed by adverse vaccine event monitoring studies of Pfizer-BioNTech vaccine in Saudi Arabia and other vaccines in general [20]. For instance, a study describing the reports within VAERS (Vaccine Adverse Event Reporting System) of the CDC from 1990 to 2016 mentioned that 80% of the anaphylaxis reports were from female participants [21]. In another study, it was stated that women (20–59 years old) were four times more likely than men to report post-vaccination allergic responses following the 2009–10 H1N1 pandemic vaccine administration [22]. Several factors might be responsible for this gender disparity in vaccine side effects. Most likely, the differences in immune responses are associated with variations in male and female hormones. It was reported in several studies that testosterone is related to immune response suppressive action in adult males, while estradiol stimulates a higher antibody response to viral infections in adult females [23,24]. This might be a plausible explanation why women experienced fatigue, headache and drowsiness more than males in the current study. Women may, therefore, be counselled before the administration of vaccines so that they would be less worried about the higher level of side effects that they might experience after vaccination.

In this study, the majority of the participants were aged 40 years and above (73%). The 166.3 million Bangladeshi population comprises 68% of people between the ages of 15 and 64 years and about 5% of people above the age of 65 years [25]. Initially, the vaccine campaign only targeted the older population (age 55 and above), as they are more susceptible to infection and disease. However, due to lesser-than-expected registrations, the age limit was promptly decreased to 40 years, which is why the majority of study participants belong to this age group. Apart from people aged 40 years and above, the government of Bangladesh also prioritised frontline workers and those belonging to certain groups of professionals for the first round of vaccines [14,26]. The results further showed that the presence and number of side effects were reported by a significantly greater number of younger adults than the older ones (*p* = 0.02). This finding is in congruence with the results of Oxford AstraZeneca’s clinical trial [11]. Similar trends were also observed in studies conducted with Covishield in Nepal [27], and with Pfizer-BioNTech vaccine in the Czech Republic and Saudi Arabia [20,28]. This phenomenon can be interpreted using the concept of immunosenescence, which refers to the decline in function of the immune system with age. The immune system may induce cytokine production that could have an inflammatory effect on the blood vessels, muscles and other tissues, causing flu-like symptoms that last for days after vaccination [20], which explains why the data in this study demonstrated a higher prevalence of fever, headache and drowsiness in younger adults compared to older ones (Table 2 and Table 3).

About two-thirds of vaccine recipients (69%) mentioned that they were confident regarding the vaccine’s efficacy, which is similar to the finding by another recent study in Bangladesh, where 67% of respondents believed that the vaccine would work against COVID-19 infection [29] (Figure 3b). The majority of the participants took vaccines due to their own will, with a workplace policy and a family member’s or friend’s influence also being prominent reasons (Figure 3a). Since a higher number of participants were from the urban and educated groups, further public awareness programs regarding the importance of vaccines may be initiated by the government.

### Study Limitations

This study focused on the short-term side effects following the first dose of the COVID-19 vaccine because very few people had received the second dose at the time of the study. Moreover, this study only described self-reported side effects from the respondents and, because of purposive sampling, the generalisability of the study’s outcome might be affected. A further study with a large and representative sample is therefore recommended.

## 5. Conclusions

Overall, the short-term side effects following the administration of the first dose of the Covishield vaccine were common but not life-threatening and the findings were consistent with the clinical trial results. Despite the higher level of side effects reported by adult women and younger populations, the vaccine had generally been well-tolerated among the different age groups of people. At the time of this study, only Covishield was being administered in Bangladesh; hence, further studies comparing both the short- and long-term side effects of the different types of COVID-19 vaccines are recommended. This might help to curtail the vaccine misconceptions and fears among the general population, encouraging more people to participate in the mass vaccination program in the coming days.

## Figures and Tables

**Table 1:** Demographic characteristics of study participants (*n* = 474).

Variable	Outcome	Frequency (*n* and %)
Gender	Male	281 (59.3)
	Female	193 (40.7)
Age	18–30 years	59 (12.4)
	31–40 years	66 (13.9)
	41–50 years	158 (33.3)
	51–60 years	123 (25.9)
	61–70 years	51 (10.8)
	More than 70 years	17 (3.6)
Educational Qualification	Illiterate	5 (1.1)
	Primary	20 (4.2)
	Secondary	43 (9.1)
	Higher secondary	68 (14.3)
	Undergraduate or higher	338 (71.3)
Occupation	Day labor	4 (0.8)
	Service holder (govt./private)	98 (20.7)
	Frontline workers	4 (0.8)
	Healthcare workers	24 (5.1)
	Teachers/students	163 (34.4)
	Business holder	59 (12.4)
	Freelancer	3 (0.6)
	Unemployed	25 (5.3)
	Housewife	93 (19.6)
	Other	1 (0.2)
Area of Residence	Rural	189 (39.9)
	Urban	285 (60.1)
Location of Vaccination Centre	Dhaka	240 (50.6)
	Chittagong	24 (5.1)
	Rajshahi	27 (5.7)
	Khulna	111 (23.4)
	Barisal	6 (1.3)
	Sylhet	3 (0.6)
	Mymensingh	8 (1.7)
	Rangpur	51 (10.8)
Body Mass Index (BMI)	<18.5 kg/m^2^ (underweight)	7 (1.5)
	18.5–24.9 kg/m^2^ (normal weight)	220 (46.4)
	25–29.9 kg/m^2^ (overweight)	177 (37.3)
	>30 kg/m^2^ (obese)	40 (8.4)
	Not known	30 (6.3)
Comorbid Conditions *	None	222 (46.8)
	Hypertension	158 (33.3)
	Diabetes	116 (24.5)
	Heart disease	40 (8.4)
	Lung disease	38 (8.0)
	Kidney disease	14 (2.2)
	Liver disease	2 (0.4)
	Cancer	1 (0.2)
	Stroke	20 (4.2)
	Other	21 (4.4)

* Indicates multiple response questions. Due to missing values, some cumulative percentages may not reach 100%. Missing values were not reported explicitly if they were below 10%.

**Table 2:** Association of the number of side effects with different demographic groups.

Variables	No Side Effects	1–3 Side Effects	4–6 Side Effects	>6 Side Effects	χ^2^ (df)	OR	95% CI	*p*-Value
	*n* (%)	*n* (%)	*n* (%)	*n* (%)	*p*-Value			
Gender								
Male	93 (33.1)	146 (52.0)	36 (12.8)	6 (2.1)	10.334 (3)	Ref.	Ref.	Ref.
Female	50 (25.9)	95 (49.2)	35 (18.1)	13 (6.7)	0.016	1.506	0.97–2.34	0.067
Age								
18–30 years	13 (22.0)	24 (40.7)	17 (28.8)	5 (8.5)	28.252 (15) 0.02	8.557	2.49–29.43	0.001
31–40 years	16 (24.2)	36 (54.5)	11 (16.7)	3 (4.5)		5.052	1.57–16.24	0.007
41–50 years	46 (29.1)	84 (53.2)	24 (15.2)	4 (2.5)		4.078	1.39–11.98	0.011
51–60 years	43 (35.0)	60 (48.8)	15 (12.2)	5 (4.1)		3.768	1.26–11.29	0.018
61–70 years	15 (29.4)	31 (60.8)	4 (7.8)	1 (2.0)		3.687	1.13–12.09	0.031
>70 years	10 (58.8)	6 (35.3)	0 (0.0)	1 (5.9)		Ref.	Ref.	Ref.

Omnibus χ^2^ (11) = 38.48 (*p* < 0.001), R^2^ = 0.079 (Cox and Snell), 0.112 (Nagelkerke).

**Table 3:** Association of individual post-vaccination side effects with gender and different age groups.

Side Effect	Total *n* (%)	Male *n* (%)	Female *n* (%)	χ^2^ (df)	18–30 Years *n* (%)	31–40 Years *n* (%)	41–50 Years *n* (%)	51–60 Years *n* (%)	61–70 Years *n* (%)	>70 Years *n* (%)	χ^2^ (df)
				*p*-Value							*p*-Value
Pain at site of injection	232 (48.9)	136 (48.4)	96 (49.7)	0.083 (1) 0.774	35 (59.3)	38 (57.6)	70 (44.3)	56 (45.5)	27 (52.9)	6 (35.3)	8.04 (5) 0.154
Feverish	134 (28.3)	79 (28.1)	55 (28.5)	0.008 (1) 0.927	21 (35.6)	16 (24.2)	43 (27.2)	33 (26.8)	18 (35.3)	3 (17.6)	4.488 (5) 0.482
Fever	115 (24.3)	65 (23.1)	50 (25.9)	0.48 (1) 0.489	16 (27.1)	19 (28.8)	50 (31.6)	23 (18.7)	4 (7.8)	3 (17.6)	15.644 (5) 0.008
Myalgia	110 (23.2)	59 (21.0)	51 (26.4)	1.892 (1) 0.169	18 (30.5)	18 (27.3)	36 (22.8)	31 (25.2)	4 (7.8)	3 (17.6)	9.718 (5) 0.084
Fatigue	83 (17.5)	41 (14.6)	42 (21.8)	4.073 (1) 0.044	16 (27.1)	15 (22.7)	22 (13.9)	21 (17.1)	8 (15.7)	1 (5.9)	8.146 (5) 0.148
Headache	65 (13.7)	28 (10.0)	37 (19.2)	8.196 (1) 0.004	16 (27.1)	11 (16.7)	21 (13.3)	14 (11.4)	2 (3.9)	1 (5.9)	15.049 (5) 0.01
Drowsiness	44 (9.3)	20 (7.1)	24 (12.4)	3.842 (1) 0.05	15 (25.4)	4 (6.1)	13 (8.2)	9 (7.3)	3 (5.9)	0 (0.0)	22.28 (5) 0.000
Dizziness	38 (8.0)	17 (6.0)	21 (10.9)	3.621 (1) 0.05	9 (15.3)	7 (10.6)	10 (6.3)	9 (7.3)	2 (3.9)	1 (5.9)	6.748 (5) 0.24
Joint pain	29 (6.1)	14 (5.0)	15 (7.8)	1.55 (1) 0.213	4 (6.8)	2 (3.0)	11 (7.0)	9 (7.3)	2 (3.9)	1 (5.9)	2.074 (5) 0.839
Nausea or vomiting	23 (4.9)	5 (1.8)	18 (9.3)	14.115 (1) 0.000	5 (8.5)	3 (4.5)	4 (2.5)	7 (5.7)	3 (5.9)	1 (5.9)	3.877 (5) 0.567
Swelling	21 (4.4)	6 (2.1)	15 (7.8)	8.586 (1) 0.003	5 (8.5)	5 (7.6)	5 (3.2)	3 (2.4)	2 (3.9)	1 (5.9)	5.687 (5) 0.338
Itching	20 (4.2)	9 (3.2)	11 (5.7)	1.765 (1) 0.184	1 (1.7)	3 (4.5)	7 (4.4)	5 (4.1)	3 (5.9)	1 (5.9)	1.438 (5) 0.92
Decreased appetite	15 (3.2)	9 (3.2)	6 (3.1)	0.003 (1) 0.954	3 (5.1)	2 (3.0)	5 (3.2)	3 (2.4)	1 (2.0)	1 (5.9)	1.576 (5) 0.904
Irritation at injection site	9 (1.9)	6 (2.1)	3 (1.6)	0.207 (1) 0.649	1 (1.7)	1 (1.5)	3 (1.9)	1 (0.8)	2 (3.9)	1 (5.9)	3.412 (5) 0.637
Diarrhea	5 (1.1)	3 (1.1)	2 (1.0)	0.001 (1) 0.974	1 (1.7)	2 (3.0)	1 (0.6)	0 (0.0)	1 (2.0)	0 (0.0)	4.862 (5) 0.433
Difficulties in breathing	4 (0.8)	3 (1.1)	1 (0.5)	0.413 (1) 0.521	0 (0.0)	1 (1.5)	1 (0.6)	0 (0.0)	1 (2.0)	1 (5.9)	7.906 (5) 0.161
Abdominal cramps	4 (0.8)	3 (75.0)	1 (25.0)	0.413 (1) 0.521	0 (0.0)	0 (0.0)	1 (0.6)	1 (0.8)	1 (2.0)	1 (5.9)	7.067 (5) 0.216
Seizure	3 (0.6)	2 (0.7)	1 (0.5)	0.068 (1) 0.794	0 (0.0)	0 (0.0)	2 (1.3)	0 (0.0)	0 (0.0)	1 (5.9)	10.36 (5) 0.066
Total	331 (69.8)	188 (66.9)	143 (74.1)	2.807 (1) 0.094	46 (78.0)	50 (75.8)	112 (70.9)	80 (65.0)	36 (70.6)	7 (41.2)	11.017 (5) 0.05

## Data Availability

The data supporting the findings of this study are available from the corresponding author, N.J., upon request.
